# Early postoperative tumor marker responses provide a robust prognostic indicator for N3 stage gastric cancer

**DOI:** 10.1097/MD.0000000000007560

**Published:** 2017-08-11

**Authors:** Qingrui Zhang, Hui Qu, Guorui Sun, Zhiqiang Li, Shuzhen Ma, Zhenxing Shi, Ensheng Zhao, Hao Zhang, Qingsi He

**Affiliations:** a Department of General Surgery; b Department of Chemotherapy, Shandong University Qilu Hospital, Jinan; c Department of General Surgery, The People's Hospital of Laoling, Dezhou, China.

**Keywords:** CA19-9, carcinoembryonic antigen, gastric cancer, N3 stage, prognosis

## Abstract

The clinical significance of tumor markers after radical gastrectomy has not been well characterized. The purpose of this study is to evaluate the prognostic value of early postoperative tumor marker normalization in N3 stage gastric cancer (GC) patients. A total of 259 N3 stage GC patients with preoperatively elevated carcinoembryonic antigen (CEA, >5 ng/mL) or carbohydrate antigen 19-9 (CA19-9, >37 U/mL) levels underwent radical gastrectomy were analyzed retrospectively. Early postoperative tumor marker response was considered as a normalization of CEA or CA19-9 levels 4 weeks after surgery. The disease-free survival (DFS) and overall survival (OS) were analyzed. N3 stage GC patients were divided into N3a (n = 157) and N3b (n = 102) groups according to the 8th TNM stage system. Early tumor marker response was identified in 96 of 157 N3a patients (61.15%) and 57 of 102 N3b patients (55.88%). In N3 stage GC patients with a tumor marker response, significant increase was observed in both DFS (25.2 months vs 12.5 months, *P* < .001) and OS (32.5 months vs 18.5 months, *P* < .001) compared with those without tumor marker response. N3b patients with a tumor marker response showed more favorable DFS (19.2 months vs 13.6 months, *P* = .019) and OS (25.8 months vs 19.0 months, *P* = .013) compared with N3a patients lacking a tumor marker response. Multivariate analysis revealed that early tumor marker response was an independent factor for DFS and OS in N3 stage GC, as well as for depth of invasion and metastatic lymph node rate (*P* < .05). Early postoperative CEA or CA19-9 normalization serves as a strong prognostic indicator in N3 stage GC. Both N3a and N3b patients with increased early postoperative tumor marker levels showed poor outcomes.

## 1 Introduction

Gastric cancer (GC), the fourth most common malignancy and the second leading cause of cancer-related death worldwide, remains one of the lethal diseases globally.^[1]^ The prognosis of the GC is poor in China due to higher rates of metastasis.^[2,3]^ Nowadays, the tumor-node-metastasis (TNM) staging system provides a standard for the classification of the anatomic extent, prediction of prognosis, as well as establishing of treatment regimen. Recently, the latest 8th edition of the American Joint Commission on Cancer (AJCC) TNM stage system subclassifies the N3 stage of GC into a N3a (7–15 regional lymph nodes metastasis) and a N3b (>15 regional lymph nodes metastasis) category. Such subclassification influences the final stages of GC.^[4]^

Carcinoembryonic antigen (CEA) and carbohydrate antigen (CA) 19-9 are the most commonly used markers for the early diagnosis of cancer. These markers have been used for the prediction of prognosis and recurrences of GC after surgery.^[5–9]^ However, rare studies have been focused on the evaluation of the prognostic significance of serum CEA and CA19-9 levels within 4 weeks after curative resection. The primary purpose of this study was to evaluate the prognostic value of postoperative serum CEA and CA19-9 in N3 stage GC patients. Our study provides an auxiliary value for the rationality of the N3 subclassification.

## 2 Patients and methods

### 2.1 Methods

A total of 383 GC patients (N3 stage) with preoperative elevation of CEA (>5 ng/mL) or CA19-9 (>37 U/mL) admitted in the Qilu Hospital of Shandong University from January 2004 to October 2011 were included. The exclusion criteria were as follows: perioperative mortality, those with incomplete clinical records (including those lost to follow-up), patients receiving palliative surgery, patients subjected to surgery with metastasectomy, and those subjected to preoperative neoadjuvant chemotherapy. Finally, 259 patients received D2 or D2+ radical gastrectomy were enrolled in this study. Patients were either subclassified as stage N3a (n = 157, 60.62%) or stage N3b (n = 102, 39.38%) according to the 8th AJCC TNM stage system. Serum CEA and CA19-9 levels were measured preoperatively and at 4 weeks after surgery. Tumor marker response was considered as a normalization of either CEA or CA19-9 levels 4 weeks after radical gastrectomy and before administration of systemic chemotherapeutic agents. Written informed consent was obtained from each patient. The study protocols were approved by the Ethical Committee, Qilu Hospital of Shandong University.

### 2.2 Determination of serum CEA and CA19-9

Peripheral blood sample was collected from each patient. Serum CEA and CA19-9 levels were measured preoperatively and 4 weeks after surgery, respectively. The determination was performed using a Roche Elecsys 2010 Immunoassay analyzer (Roche, Penzberg, Germany) using the electrochemiluminescence method.

### 2.3 Follow-up

Follow-up was conducted 4 weeks after surgery, and then was conducted every 1 to 3 months in the first year, every 3 to 6 months in the next 2 years, and every 6 months thereafter until 5 years postoperatively. The median duration of follow-up at the cutoff date was 26.5 months (range = 5.3–107.0 months). During the follow up, the patients were required to receive physical examination, determination of serum CEA and CA19-9 levels, chest radiogram and abdominal ultrasound (US). Diagnostic imaging such as abdominopelvic computer tomography (CT), esophagogastroduodenoscopy (EGD), or positron emission tomography-CT was performed if necessary. Recurrence was evaluated by clinical symptoms, imaging findings, histological biopsy, or findings at reoperation.

### 2.4 Statistical analysis

Data analysis was performed using SPSS 22.0 software. Clinicopathologic features were analyzed using Chi-square test. The median disease-free survival (DFS) and overall survival (OS) were estimated according to the Kaplan–Meier method, and log-rank test was performed for the comparison. Variables that significantly affected the DFS and OS were investigated by multivariate analysis according to the Cox regression model. *P* < .05 was considered to be statistically significant.

## 3 Results

### 3.1 Clinicopathologic features

Clinicopathologic features of 259 patients (male: 171; female: 88) of stage N3 are summarized in Table 1. The median age was 57 years (23–86 years). Based on the 8th AJCC TNM stage system, 157 patients (60.62%) were categorized into N3a stage, while 102 patients (39.38%) were categorized into N3b stage. Early postoperative serum CEA or CA19-9 normalization was observed in 153 patients (59.07%). No significant differences were noticed in the sex, age, N3 subclassification, depth of invasion, tumor size, tumor location, type of surgery, as well as lymph vessel invasion and differentiation between the 2 groups. Patients with no tumor marker response showed metastatic lymph node ratios of ≥0.4 compared with those with tumor marker response (*P* = .025).

**Table 1:**
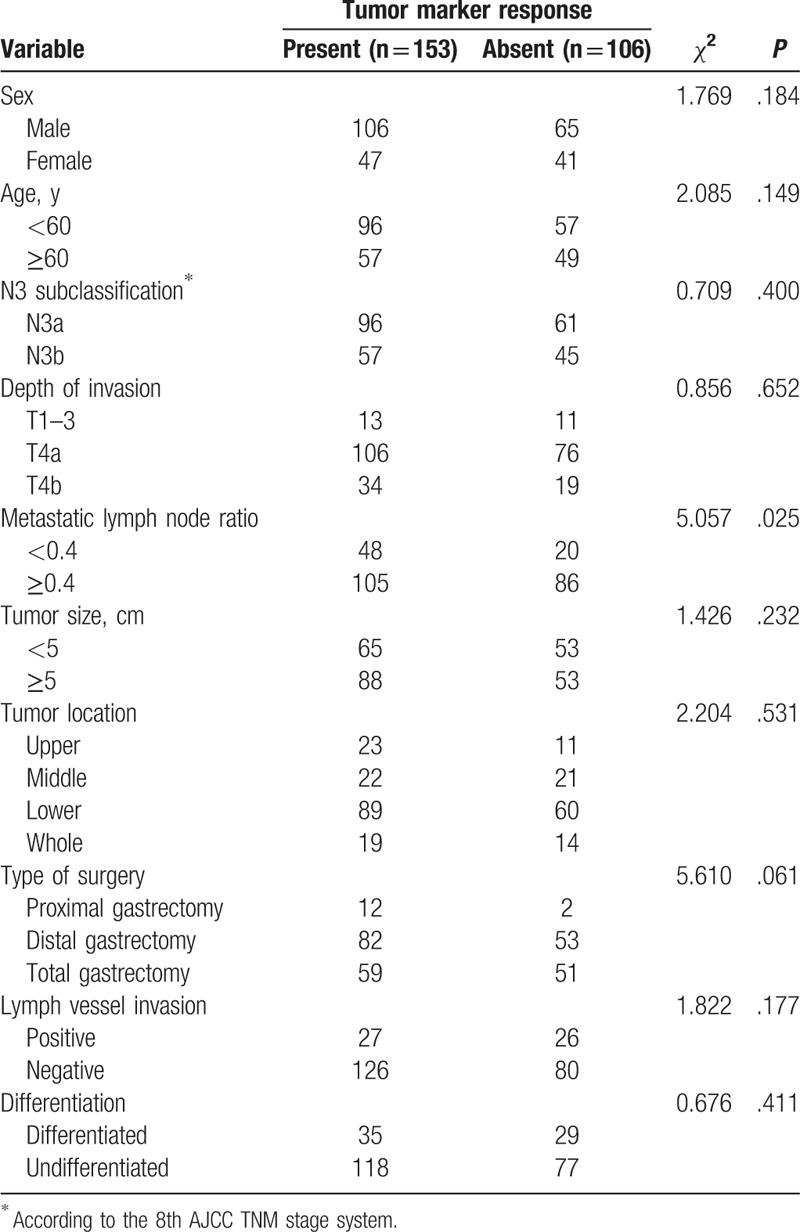
Clinicopathologic features in N3 stage GC patients by tumor marker response.

### 3.2 Comparison of survival outcomes in patients with or without tumor marker response

**Figure 1:**
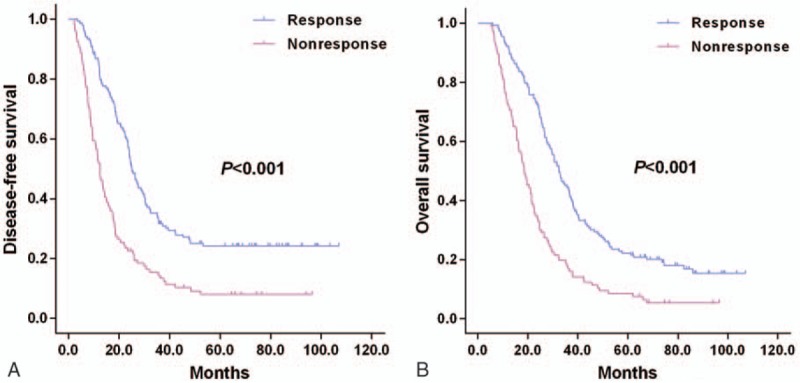
Survival outcomes of tumor marker response and nonresponse. Kaplan–Meier curves of DFS (A) and OS (B).

**Figure 2:**
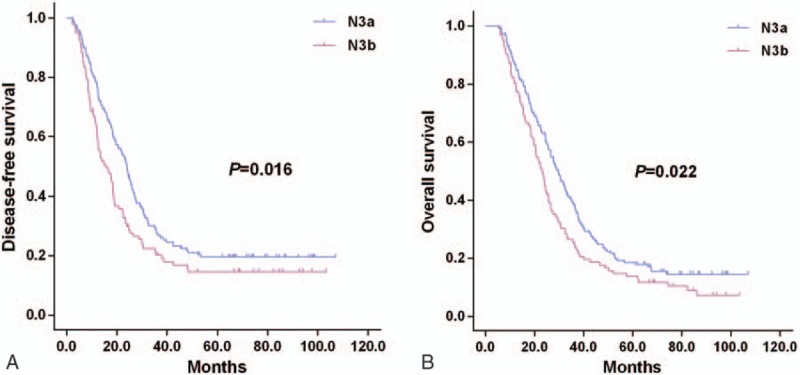
Survival outcomes of N3a and N3b subclassifications, Kaplan–Meier curves of DFS (A) and OS (B).

**Figure 3:**
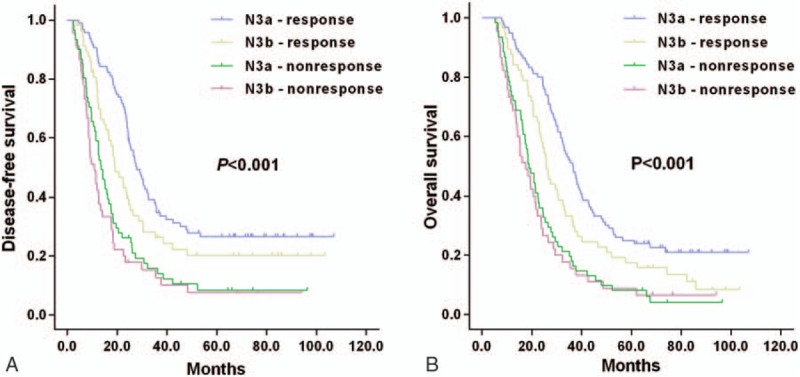
Survival outcomes of tumor marker response and nonresponse, divided into N3a and N3b response, Kaplan–Meier curves of DFS (A) and OS (B).

Survival curves of GC patients are depicted in Fig. 1 based on the tumor marker response status. GC patients at N3 stage with a tumor marker response showed favorable outcomes than those lacking of a tumor marker response (nonresponse) in terms of DFS (25.2 months vs 12.5 months, *P* < .001, Fig. 1A) and OS (32.5 months vs 18.5 months, *P* < .001, Fig. 1B). N3a patients showed significantly higher DFS (24.0 months vs 15.5 months, *P* = .016, Fig. 2A) and OS (29.5 months vs 22.9 months, *P* = .022; Fig. 2B) than those of N3b patients. Interestingly, the N3b patients with a tumor marker response showed significantly better outcomes than N3a patients lacking a tumor marker response in terms of DFS (19.2 months vs 13.6 months, *P* = .019, Fig. 3A) and OS (25.8 months vs 19.0 months, *P* = .013, Fig. 3B), respectively. No statistical differences were noticed in the DFS (13.6 months vs 10.8 months, *P* = .365, Fig. 3A) or OS (19.0 months vs 17.6 months, *P* = .697, Fig. 3B) between patients at N3a and N3b stages lacking a tumor marker response.

### 3.3 Recurrence site and rate

The initial recurrence sites and rates are listed in Table 2 according to early postoperative serum tumor marker levels. No statistical differences were identified in the initial recurrence in the liver, lung, bone, lymph nodes, and peritoneum anastomosis, as well as other organs in patients with response than those without. The recurrence rate within 1 year after surgery was significantly higher in the tumor marker nonresponse patients compared to the response patients (53.13% vs 21.43%, *P* < .001).

**Table 2:**
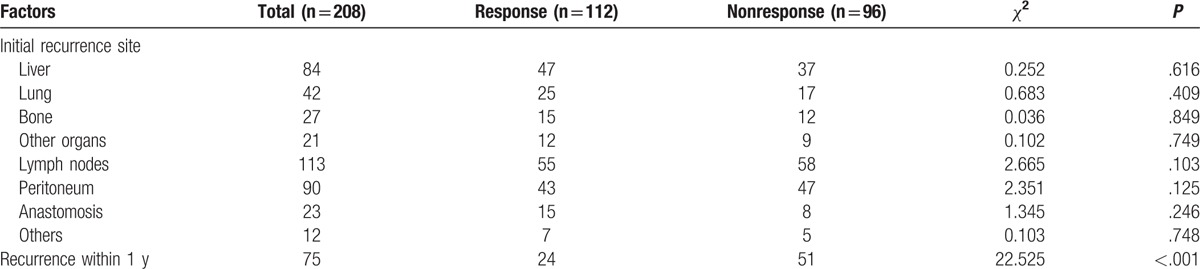
Initial recurrence site and recurrence rate within 1 year.

### 3.4 Prognosis factors

Prognostic factors for DFS and OS were analyzed using multivariate analysis according to Cox regression model. Univariate analysis showed that several factors were risk factors for DFS including tumor marker response (*P* < .001), N3 subclassification (*P* = .016), depth of invasion (*P* = .001), metastatic lymph node rate (*P* < .001), tumor location (*P* = .025), type of surgery (*P* = .002), and lymph vessel invasion (*P* = .047, Table 3). Meanwhile, the risk factors for OS were tumor marker response (*P* < .001), N3 subclassification (*P* = .022), depth of invasion (*P* = .001), metastatic lymph node rate (*P* < .001), tumor size (*P* = .011), tumor location (*P* = .034), and type of surgery (*P* = .004). According to the multivariate analysis, several independent factors for DFS and OS were identified including tumor marker responses (*P* < .001), as well as for depth of invasion (*P* = .001) and metastatic lymph node rate (*P* < .001).

**Table 3:**
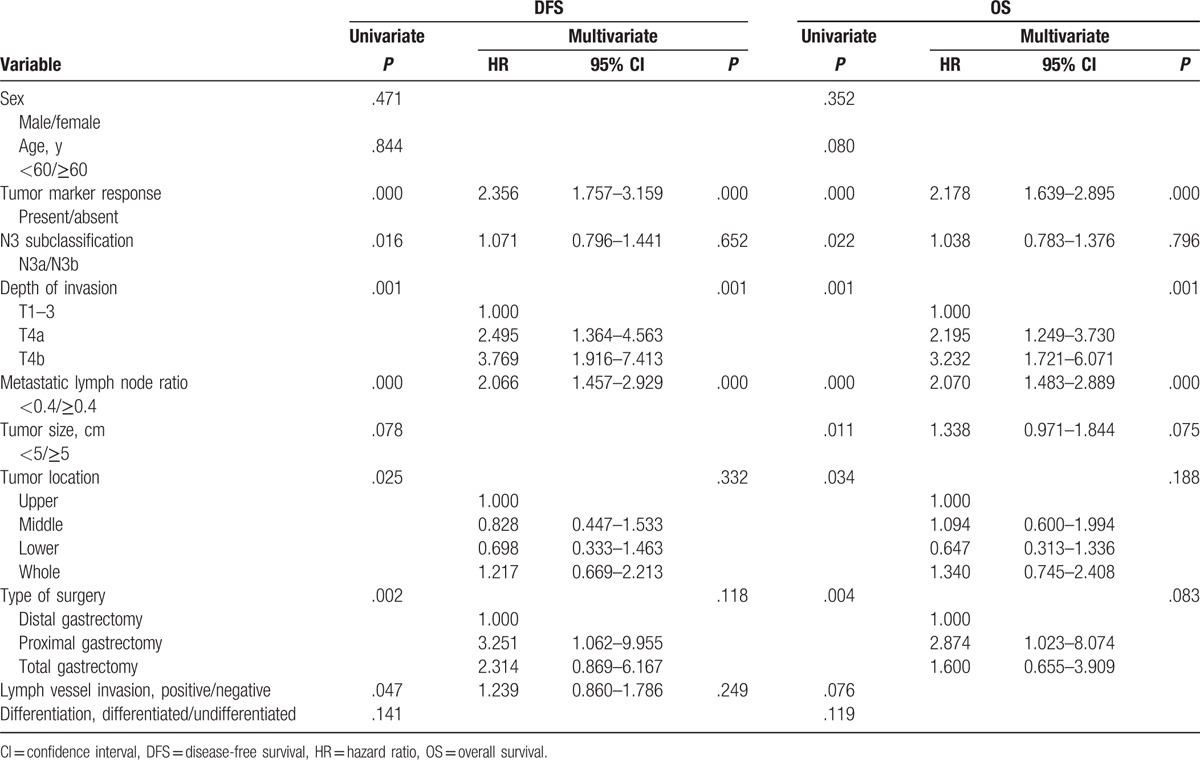
Univariate and multivariate analysis for DFS and OS in N3 stage GC patients.

## 4 Discussion

N3 stage GC has been recognized as an advanced malignancy with a poor prognosis, even after radical resection.^[10]^ The prognosis of GC patients vary greatly even within patients at the same TNM stage.^[11]^ Our study showed that early CEA or CA19-9 normalization after radical gastrectomy was a prognostic factor for N3 stage GC, which was particularly relevant for patients with high preoperative levels of tumor markers. N3b patients with tumor marker response showed a better prognosis compared to that of N3a patients lacking tumor marker response. We assumed that early postoperative CEA or CA19-9 may reflect the radical extent of surgery in N3 stage GC patients.

Several studies demonstrated that elevation of preoperative CEA and CA19-9 could serve as independent prognostic factors for patients underwent radical surgery.^[7,12,13]^ Nam et al^[14]^ reported that early postoperative tumor marker levels might be used for the prediction of survival of GC patients. However, early stage GC patients showed lower recurrence sensitivities of postoperative tumor markers compared with that in advanced GC.^[15]^ At present, the prognostic impact of tumor markers in N3 stage GC patients remains elusive. N3 stage GC patients are more likely to show abnormal preoperative tumor marker levels.^[16–18]^ According to our experience, N3 stage GC patients with elevated tumor markers after radical gastrectomy tended to show poor outcomes. Therefore, a precise determination of the prognostic value of tumor markers in N3 stage GC would be of substantial importance in clinical practice. To our best knowledge, our study firstly reported the prognostic impact of the early postoperative serum CEA and CA19-9 levels in N3 stage GC patients.

In this study, the prognostic value of the postoperative serum tumor marker response in patients with N3 stage GC was analyzed retrospectively. Postoperative serum CEA and CA19-9 concentrations were initially measured 4 weeks after surgery to reduce the potential for any confounding effects of adjuvant treatment on postoperative serum tumor markers levels. According to our results, 106 patients (40.93%) showed aberrant serum CEA or CA19-9 levels. N3 stage GC patients in the tumor marker nonresponse group showed lower DFS and OS than those in the response group. Tumor markers were expressed in the process of tumorigenesis and progression and may indicate the presence of a neoplasm as well as the relative malignancy burden and aggressive nature of the biological response.^[19]^ Early postoperative nonresponse tumor markers were more likely to be found in patients with higher tumor burdens, and those with the potency for increased prevalence of micrometastases.^[20]^ In cases of the fact that serum tumor markers failed to get in the normal range within 4 weeks postoperatively, the possibility of an incomplete resection of the primary tumor should be considered with or without the presence of micrometastases.^[14,21]^ The recurrence rate within 1 year after surgery was significantly higher in nonresponse (53.13%) as compared with response (21.43%) patients. Therefore, as the half-life of CEA and CA19-9 was short,^[22,23]^ we cautiously suggested that an early normalization of tumor markers might reflect the efficiency of radical operation and an elimination of the invasive potential of the cancer. N3 stage GC patients, particularly those with no tumor marker response at the early stage, should be closely followed up together with administration of intensive chemotherapy.

The extent of lymph node metastasis was considered to be the most important independent prognostic factor for GC.^[16,24]^ In this study, statistical difference was noticed in the outcomes between N3a and N3b patients, which was also reflected in the TNM stage of the latest 8th AJCC edition.^[4]^ When N3 patients were stratified into 4 groups based on early postoperative tumor marker status and N3 subclassification, we interestingly found that N3b patients with a tumor marker response showed more favorable outcomes than N3a patients lacking a tumor marker response. However, for both N3a and N3b patients lacking a tumor marker response, the DFS and OS were similar and presented in a decline tendency. Therefore, more attention should be paid to the postoperative management of N3 stage GC patients based on their early postoperative tumor marker status, rather than simple stratification based on the AJCC TNM staging system.

Identification of prognostic factors may contribute to the prediction and improvement of outcomes in GC patients. As an important prognostic factor, depth of tumor invasion is included in the AJCC TNM stage system.^[25]^ Moreover, metastatic lymph ratio has also been reported as a promising prognostic indicator in GC.^[26,27]^ Our study revealed that early tumor marker response, depth of invasion, and metastatic lymph node ratio were the independent prognostic factors for DFS and OS according to multivariate analysis. Therefore, these factors can serve as useful parameters to predicate the prognosis of N3 stage GC patients after radical gastrectomy.

Indeed, there are limitations in our study. Firstly, our study was retrospective with a relatively small sample size. Secondly, we only assessed 2 markers in our analysis while CA72-4 that was considered as a useful follow-up tumor marker after gastrectomy was not included. Finally, it is not possible to apply our results to all stages of GC patients as it is still a challenge to identify the presence of high levels of preoperative tumor markers in early stage GC patients.

## 5 Conclusions

In conclusion, early postoperative serum tumor marker responses can serve as a strong prognostic indicator for N3 stage GC. Early postoperative serum CEA and CA19-9 levels may contribute to the prediction of prognosis, which can better reflect the status of the curative resection. During the postoperative follow-up, careful attention should be paid to N3 stage GC patients based on their postoperative tumor marker levels, rather than simply stratifying patients based on the AJCC TNM staging system. Meanwhile, N3a and N3b patients with elevation of early postoperative tumor marker levels should be closely followed up together with administration of intensive chemotherapy.
